# Cell Hypertrophy: A “Biophysical Roadblock” to Reversing Kidney Injury

**DOI:** 10.3389/fcell.2022.854998

**Published:** 2022-03-03

**Authors:** Angelo Michele Lavecchia, Kostas Pelekanos, Fabio Mavelli, Christodoulos Xinaris

**Affiliations:** ^1^ Laboratory of Organ Regeneration, Department of Molecular Medicine, Istituto di Ricerche Farmacologiche Mario Negri IRCCS, Centro Anna Maria Astori, Bergamo, Italy; ^2^ Independent scholar, Nicosia, Cyprus; ^3^ Department of Chemistry, University of Bari Aldo Moro, Bari, Italy

**Keywords:** hypertrophy, regeneration, metabolism, podocytes, proximal tubular epithelial cells, kidney injury

## Abstract

In anamniotes cell loss can typically be compensated for through proliferation, but in amniotes, this capacity has been significantly diminished to accommodate tissue complexity. In order to cope with the increased workload that results from cell death, instead of proliferation highly specialised post-mitotic cells undergo polyploidisation and hypertrophy. Although compensatory hypertrophy is the main strategy of repair/regeneration in various parenchymal tissues, the long-term benefits and its capacity to sustain complete recovery of the kidney has not been addressed sufficiently. In this perspective article we integrate basic principles from biophysics and biology to examine whether renal cell hypertrophy is a sustainable adaptation that can efficiently regenerate tissue mass and restore organ function, or a maladaptive detrimental response.

## Introduction

In response to injury, anamniotes–i.e., fish and amphibians–compensate for cell loss through proliferation and differentiation, which allows them to partially or completely regenerate tissues and organs (Brockes and Kumar, 2008; Gemberling et al., 2013). Adult mammals, on the other hand, have lost this capacity, partially due to endothermy (Hirose et al., 2019; Nguyen et al., 2021), and because of the cumulative structural and functional complexity of organs (Jaźwińska and Sallin, 2016). In response to injuries, the highly specialised and terminally differentiated cells of mammalian organs, in order to cope with the increased workload, typically undergo hypertrophy and/or polyploidisation. This allows complex organs, such as the mammalian kidney, to deal with the increased workload without significantly compromising structure and function, which makes this trait vital for tissues where the architecture is implicitly and linearly correlated with function.

In response to injury, podocytes undergo polyploidy by DNA synthesis and G2/M arrest, and grow in size until they eventually coat the areas that were depleted (Najafian et al., 2011; Lasagni et al., 2013; Benedetti et al., 2019). Interestingly, recent studies have proposed that detached podocytes can be replaced by neighbouring podocyte progenitors. Two podocyte progenitor pools have been proposed: parietal epithelial cells (PECs) (Sagrinati et al., 2006; Appel et al., 2009) and cells of renin lineage (CoRL) (Pippin et al., 2015; Eng et al., 2018). Although the common developmental programme of PECs and podocytes (Grouls et al., 2012; Meyer-Schwesinger, 2016) can partially justify the ability of PEC progenitors to transdifferentiate into podocytes, their substantial involvement in glomerulus repopulation has been inconsistent (Berger et al., 2014; Wanner et al., 2014). Furthermore, a number of studies have suggested not only that PECs are not involved in podocyte regeneration, but that they contribute to the development of crescent, glomerulosclerosis and extracapillary proliferation (Smeets et al., 2004; Smeets et al., 2011; Gaut et al., 2014; Sakamoto et al., 2014). Studies in CoRL have also provided conflicting results and, most importantly, have not shown through which developmental programme CoRL can transdifferentiate into podocytes (Meyer-Schwesinger, 2016). Finally, very recent studies–which support earlier evidence that renin cells are pericytes (Brunskill et al., 2011; Stefanska et al., 2016)—have shown that CoRL transdifferentiated exclusively to mesangial cells (Steglich et al., 2020). We could consider several variables in any attempt to explain these inconsistencies, such as intrinsic methodological differences in species and models, the timing of analysis, and the severity of injury/disease–but so far, the main recovery mechanism of injured glomeruli seems to be podocyte hypertrophy.

Hypertrophy has also been associated with tubular injury–the most common cause of acute kidney injury (AKI). AKI is typically reversible because the mammalian renal tubule has conserved an extraordinary capacity for regeneration. The dominant dogma is that tubular regeneration after AKI can be achieved thanks to the surviving epithelial cells, which dedifferentiate, proliferate, migrate, and re-differentiate into newly generated tubular cells (Vogetseder et al., 2008; Chang-Panesso et al., 2019). However, recent studies have proposed that renal functional recovery after AKI occurs mainly through tubular cell hypertrophy, and with a minimal contribution from resident progenitor cells (Lazzeri et al., 2018). While the conundrum of tubular repair/regeneration is still being investigated vigorously, the presence of hypertrophy in proximal tubules has long been demonstrated in several models (Wolf et al., 1993; Kobayashi et al., 1995), indicating that under certain conditions (in terms of the type, severity and duration of the stress) proximal tubular epithelial cells (PTECs) undergo hypertrophy to replenish the lost tissue mass. Either if we accept that dedifferentiated proximal tubule epithelial or intratubular progenitor cells are responsible for cell replacement, hypertrophy may depend on stimulus intensity: mild stimuli may cause reversible damage that is completely repaired through proliferation, while stronger stimuli that cause extensive cell depletion may induce hypertrophy of surviving cells to grow and restore critical tissue mass.

The above findings suggest that podocytes and proximal tubular cells share some common mechanisms of response to injury, including hypertrophy and possibly a limited contribution by progenitor cells. One crucial question that has not been investigated sufficiently is whether renal cell hypertrophy is a long-standing repair/regeneration adaptation. If so, tissue mass replacement and organ recovery could indeed be mediated by hypertrophy, which would otherwise be a maladaptation and incompatible with reversibility after injury and complete recovery. Here we will try to address this question by examining podocyte and PTEC hypertrophy from the biophysic and energetic perspectives.

## Why Does the Size of Cells Matter?

A cell can be considered an open chemical reactor that uses the energy input from the environment in the form of light radiation, nutrients, osmotic gradient and electrostatic potential to sustain an internal metabolic network that transforms nutrients into all the cell constituents, and at the same time produces heat, osmotic work and by-products. These are in turn exchanged with the environment in order to maintain homeostasis and keep a tissue/organ at a constant temperature.

Now we will examine whether hypertrophy of surviving cells can lead the organ to a better or poorer homeostatic condition compared to the original condition.

**FIGURE 1:**
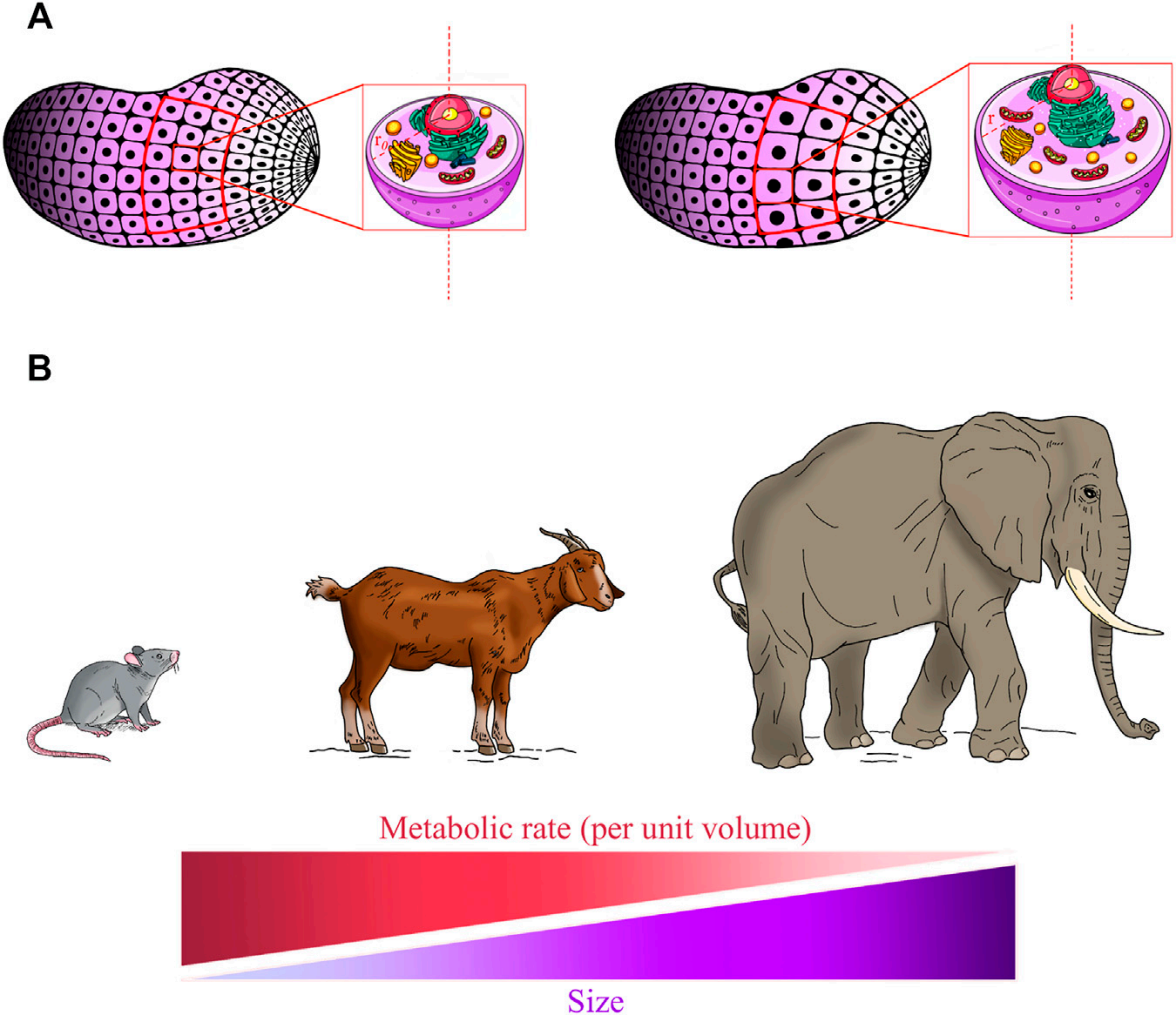
Living system’s size affects homeostatic balance and metabolic rate. **(A)** An ideal condition in which an organ section consists of “*n*” identical spherical cells with an average radius “*r*_*0*_” (left panel, red frame). In response to injury, the surviving cells grow to radius “*r*” (right panel, red frame) to compensate for tissue loss. As the cell grows bigger, the volume increases more rapidly (r^3^) than does the surface area (r^2^), and so the relative amount of surface area available to pass materials to a unit volume of the cell decreases. As the tissue section remains constant, the total membrane surface of the cells is reduced and this results in the decreased efficiency of the tissue in preserving homeostasis. Moreover, intracellular transport distances and diffusion times of oxygen and nutrients are increased and the metabolic rate decreased, negatively affecting overall cell efficiency in conserving cellular homeostatic conditions. **(B)** Allometric laws are among the most fundamental features of life and it is believed that they can be applied to all size scales. Allometric scaling of the metabolism predicts that the metabolic rate per mass unit declines with the increase in size in living systems.

For the sake of simplicity, we will consider a section of an organ with a volume *V* as an assembly of cells in a spherical shape (Figure 1A) with an average radius *r*_*0*_. Therefore, if the cells are identical, the number of cells that form the organ section can be estimated using the relationship:

$$V=n\left(\frac{4}{3}\pi r_{0}^{3}\right)$$ (1)

while the total surface area of the cell membrane will be:

$$A_{0}=n\left(4\pi r_{0}^{2}\right)$$ (2)

If we assume that a number *k* of cells is now lost, to maintain the volume of the organ section constant the neighbouring cells must grow and occupy the empty volume. Therefore, now each cell will exhibit a larger radius *r* so the volume of this section is:

$$V=\left(n-k\right)\frac{4}{3}\pi r^{3}$$ (3)

From relations Eqs 1, 3 we deduce:

$$r={\left(\frac{n}{n-k}\right)}^{\frac{1}{3}}r_{0}$$ (4)

So now the total area *A* of the cell membrane will be:

$$A=\left(n-k\right)4\pi r^{2}$$ (5)

Dividing *A*_0_/*A* we get:

$$\frac{A_{0}}{A}={\left(\frac{n}{n-k}\right)}^{\frac{1}{3}}$$ (6)

Since $\left(\frac{n}{n-k}\right)>1$ we deduce that *A < A*_0_, that is, while the total volume of the organ section *V* remains constant, which corresponds to the overall volume of the cells, the total membrane surface of the cell assembly is reduced.

It is important to highlight that both the substrate transport and the heat transfer in a cell occur through its membrane towards and from the environment, and the rate of these processes is therefore proportional to the cell surface area. Hence, a section of an organ which consists of a high number of small cells is more efficient in conserving a homeostatic condition than the same section with a lower number of larger cells (Figure 1A).

Scaling down to the level of a single cell, it is important to underline that cells need catalysts (enzymes), which enable chemical reactions to take place in a reasonable timeframe, to maintain the organ’s function and structure in a condition of homeostatis (Himeoka and Kaneko, 2014). From this point of view there are two key factors that enable cells to achieve a stationary condition within a reasonable timeframe: (i) nutrient flow through the membrane, in order to have enough energy input and (ii) maintenance of catalyst abundance.

Assuming that when the cell grows in volume it maintains a constant density of enzymes–that is, the number of enzymes scales with the volume–this means the enzymatic rate per unit volume remains the same. Thus, if a cell increases its volume by a factor of $\lambda_{v}\equiv \left(\frac{v}{v_{0}}\right)={\left(\frac{r}{r_{0}}\right)}^{3},$ *v*_o_ being the initial volume and *r*_0_ being the initial radius, then the nutrient flow *f* through the membrane should grow by at least the same factor:

$$f∼\lambda vf_{0}$$ (7)

to sustain the internal metabolism at the same homeostatic condition. On the other hand, if cells keep the spherical shape, the area of the membrane grows only by a factor of $\lambda_{A}\equiv \left(\frac{a}{a_{0}}\right)={\left(\frac{r}{r_{0}}\right)}^{2}$ with *a*_0_ being the initial surface area of the single cell.

$$\frac{\lambda_{v}}{\lambda_{A}}=\frac{r}{r_{0}}>1$$ (8)

Since the flow through a surface is proportional to the surface area, we can claim that as a cell grows in volume, it is harder to satisfy relation Eq. 7 because of relation Eq. 8 and thus the cell cannot remain as metabolically efficient, under conditions of homeostasis, as the original smaller cells.

Each internal region of the cell must be served (in terms of nutrients and oxygen) by a part of the cell surface. As the cell grows bigger, its internal volume enlarges and the cell membrane expands; however, the volume increases more rapidly (r^3^) than does the surface area (r^2^), and so the relative amount of surface area available to pass materials to a unit volume (surface-to-volume ratio) of the cell steadily decreases. Thus, less and less material will be able to cross the membrane quickly enough to accommodate the increased cellular volume.

**FIGURE 2:**
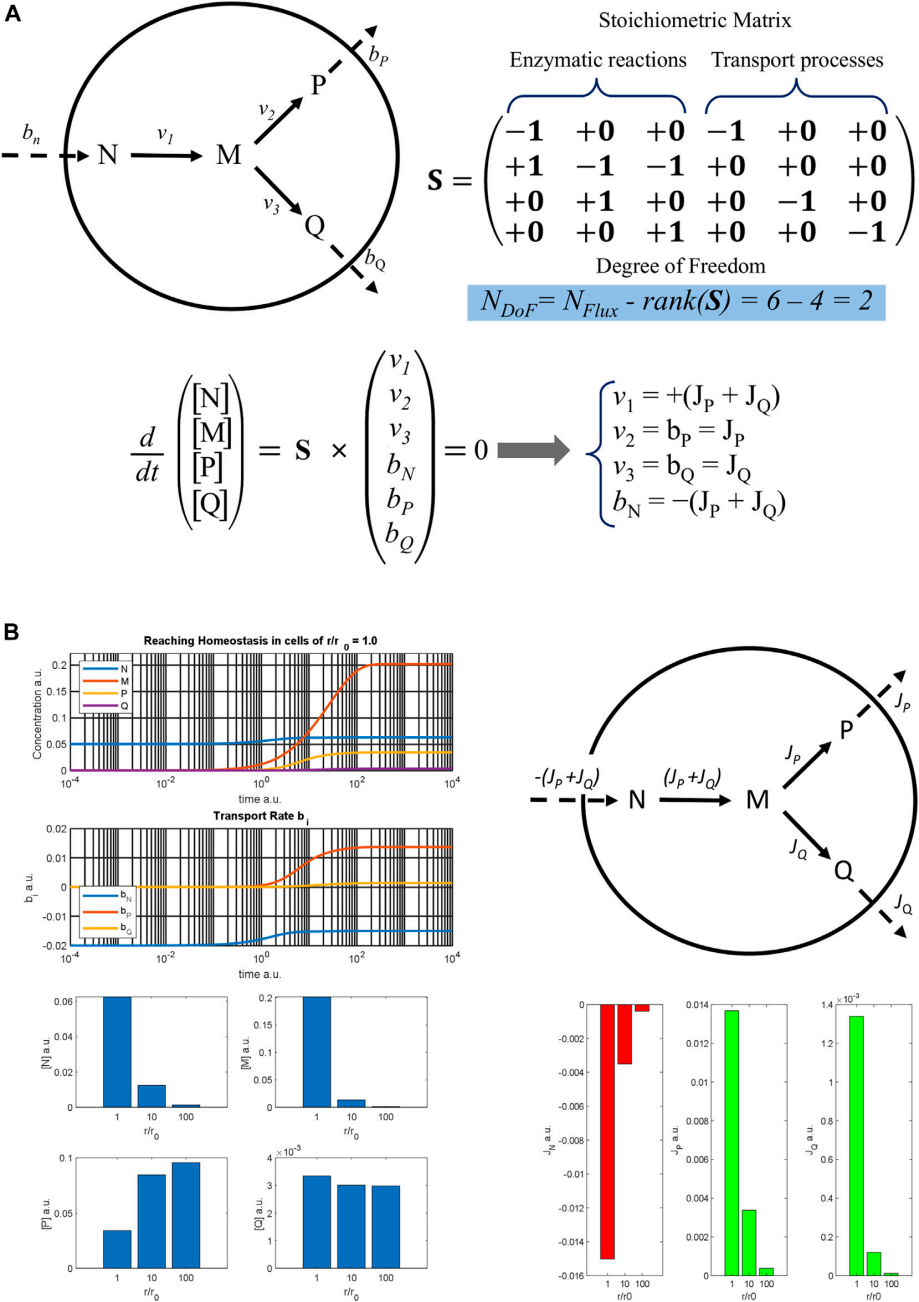
Metabolic model of a three-enzyme pathway **(A)** A three-enzyme metabolic pathway model is reported with the result of the flux balance analysis. The rank of the stoichiometric matrix **S** is 4, so that the degree of freedom of the algebraic system obtained by applying the stationary condition for homeostasis is 2. Therefore, only two fluxes, *J*_*P*_ and *J*_*Q*_ must be determined to know the stationary rates of all the processes at homeostasis. **(B)** On the top left, the time courses of the metabolite concentrations and of the rates of the transport processes are reported against time, showing a cell with a normal radius *r*_*0*_ as it reaches homeostasis; on the top right, a metabolic map with the flux distribution at homeostasis is sketched; on the bottom left, stationary concentrations of different metabolites are shown, and on the bottom right the values of the fluxes are reported as diverse histograms for cells with an increasing ratio of *r/r*_*0*_.

To better illustrate this effect, a simple metabolic pathway involving three enzymes and supported by the flow of nutrient N from the outside is presented in Figure 2A. N diffuses across the cell membrane at rate *b*_N_ and is then converted to the intermediate M by the first enzyme with rate *v*_1_. In turn M can be converted into metabolites P or Q by the second and the third enzymes, with rates *v*_2_ and *v*_3_, respectively, according to the metabolic map. It is assumed that P and Q diffuse through the cell surface with *b*_P_ and *b*_Q_ transport rates, while the cell membrane is impermeable to the intermediate M. Analysing the stoichiometric matrix, the degree of freedom of this metabolic pathway is 2, which means that only two fluxes (i.e., process rates in stationary conditions) must be determined to define all the process velocities at homeostasis. The distribution of the fluxes on the metabolic map is reported at the top right of Figure 2B, showing the input flux of the nutrient N, negative value, and the two outflows of the metabolites P and Q, positive values.

Based on this metabolic map, a dynamic model was implemented assuming a reversible Michaelis-Menten kinetic mechanism for each enzyme and using some hypothetical parameters. The time trend of metabolite concentrations and transport rates is reported at the top left of Figure 2B. The graph shows that the system reaches a stable stationary condition, which is cellular homeostasis. Assuming a spherical shape and increasing the cellular radius *r*, the dynamic simulation was repeated for *r*/*r*_0_ ratios equal to 10 and 100 and the homeostatic values obtained are reported in the bar graphs below. As can be seen, the outflows flux *J*_P_ and *J*_Q_ decrease markedly when the *r*/*r*_0_ ratio increases, as does the absolute value of the input flux |JN| = (*J*_P_ + *J*_Q_). This determines the reduction of the steady concentration both of N and M. On the other hand, the stationary concentration values of P and Q change quite little being the reduction of the enzymatic rate, due to the decrease in [M], balanced by the reduction of the outcoming fluxes. Therefore, both the efficiency and the composition of the cell in homeostasis are perturbed by the hypertrophic growth of the cell. Moreover, the *J*_P_/*J*_Q_ ratio also changes, going from 10.2, when *r*/*r*_0_ = 1, to 32.0 when *r*/*r*_0_ = 100, also exhibiting a different flux distribution at homeostasis.

It is worth mentioning that in this simple model we have not taken into account molecular diffusion in the cellular milieu by assuming these very fast processes. Indeed, the intracellular transport distances and diffusion times of oxygen and nutrients increase too, and the transport of metabolites and oxygen becomes limiting. As such, the large cells have a decreased size-normalised metabolic rate, as measured by oxygen consumption (Rubner, 1883; Kleiber, 1932). This phenomenon, known as allometric scaling of metabolism, is one of the most fundamental features of life, and is believed to apply to all size scales of biological systems (West et al., 2002; West and Brown, 2005) (Figures 1A,B). The size-dependent limitations in nutrient and oxygen transport impose a limit on the metabolism and thus on cell size, and as such the optimal cell size must be at a point where the metabolic (i.e., mitochondrial functionality) and cellular fitness (i.e., viability) are maximised (Miettinen and Björklund, 2016; Miettinen and Björklund, 2017). This size must perfectly reflect the optimal size for its functions (Ginzberg et al., 2015). Hence, the third claim is that the hypertrophied version of our hypothetical cell type–if it grows isometrically (in terms of cellular and organelle content; Figure 1A)—would in principle perform with a suboptimal metabolism, function and viability.

Based on the above claims, we can support the net thesis that for a given function or functions, a cell type has a biophysically optimal size where metabolic efficiency and cell-size-dependent homeostatic conditions are maximised. Any deviation from this size would theoretically reflect substantial alterations to cellular and organismal homeostasis.

## Is Hypertrophy Biophysically and Metabolically Efficient for Podocytes and PTECs?

For podocytes and PTECs–the functions of which entail adequately covering a given area (basement membranes)—functionality is linearly correlated with their size and must be maximised at a biophysically optimal cell size. Therefore, hypertrophied podocytes and PTECs operate under kinetically unfavourable conditions and at a suboptimal metabolic rate, which makes hypertrophy incompatible with complete organ repair/regeneration and reversible injuries. As such, hypertrophy cannot be used to explain conditions where kidney function is largely restored, like typical AKI.

One could argue against these claims by maintaining that profound cellular remodelling (in terms of shape and organelles) takes place during hypertrophy, as happens during cell proliferation, to allow hypertrophic cells to perform optimally without having irreversible effects on homeostasis. It is believed that proliferating cells overcome this metabolic barrier by undergoing allometric mitochondrial remodelling (Miettinen and Björklund, 2016; Miettinen and Björklund, 2017). Although cellular protein and organelle content increases isometrically (linearly; as in the Figure 1A) across a wide range of cell types (Savage et al., 2007), increased mitochondrial connectivity can alleviate energy transport limitations, enabling a higher metabolic rate and larger cell size, and thus allowing cells to grow and divide. In essence, this is a transient condition that allows proliferating cells to give rise to 2 daughter cells that will inherit their mother’s low metabolic rate and, as they grow, to reset their mitochondrial activity to match their size (Miettinen and Björklund, 2016; Miettinen and Björklund, 2017). Likewise, mitochondrial dynamics can explain the transient growth of skeletal muscle in response to exercise, regeneration and the growth of amphibian limbs and other cases of physiological growth (Drake et al., 2016; Qin et al., 2021). However, unlike these cell types, hypertrophied podocytes and PTECs maintain a biophysically suboptimal metabolism and size, and a permanently inefficient homeostatic status. In addition, podocytes have relatively low density of mitochondria compared to PTECs (Na et al., 2021) and their metabolism seems to be mainly based on glycolysis (Brinkkoetter et al., 2019). Although it remains intensely debated what the actual source of ATP in podocytes is (Ozawa et al., 2015; Gujarati et al., 2020; Audzeyenka et al., 2021; Na et al., 2021), based on the above premises we could safely deduce that presumptive increased mitochondrial connectivity could not have a substantial impact on metabolic functionality and cellular fitness (even if such mitochondrial remodelling existed in podocytes). On the other hand, PTECs have a high number of mitochondria and their metabolism relies mostly on beta-oxidation of fatty acids under physiological conditions (Faivre et al., 2021). During AKI PTECs undergo rapid fragmentation of mitochondria (Brooks et al., 2009; Xiao et al., 2014; Clark and Parikh, 2020) and a metabolic shift toward glycolysis (Lan et al., 2016; Faivre et al., 2021)—which again undermines the hypothesis of mitochondria remodelling. Most importantly, the transient metabolic switch seems to be indispensable during the early phases after injury and is reversed in normally recovering tubules but, if persistent, could lead to mitochondrial dysfunction and failure in tubular repair (Lan et al., 2016). Hence, from a biophysical and energetic perspective, although renal cell hypertrophy enables tissue mass replacement, it is a roadblock to complete repair and recovery.

It has been suggested that hypertrophy is an adaptive evolutionary response that allows for tissue growth, repair and regeneration in various species and taxa. For example, mammary epithelial cells through polyploidization and hypertrophy can increase DNA and transcriptional/translational capacity to rapidly increase secretion of their products (Rios et al., 2016), while liver co-ordinately combines proliferation, stem/progenitor cell differentiation and polyploidization-mediated hypertrophy to completely restore tissue mass and function. The short lifespan and the ability of liver cells to undergo reductive mitoses and proliferate, as well as the nature of their functionality, make liver hypertrophy an excellent adaptive strategy for coping rapidly with increased workload demands or injuries (Donne et al., 2020; Matsumoto et al., 2020). On the other hand, in organs with very limited regenerative potential and strong interdependence between architecture and function, as the heart, although cell hypertrophy can temporally normalize wall tension and function, when is persistent and especially when accompanied by adoption of a low energy profile, becomes maladaptive and often leads to failure and death (Frey and Olson, 2003; Pantos et al., 2008).

Is hypertrophy an adaptation or maladaptation in the mammalian kidney? Unlike amphibians and fish, which can regenerate whole organs, terrestrial animals cannot regenerate whole nephrons *de novo* without influencing homeostasis, because of the complexity and interdependence of architecture and function. Therefore, natural selection would have favoured genes that block mitosis and orchestrate a metabolic and structural profile that would allow for growth without cytokinesis (Mourouzis et al., 2020; Mantzouratou et al., 2022). This adaptation in turn improved the reproductive value of individual organisms that were capable of renal cell hypertrophy at least until the end of their reproductive lives. After this period, the detrimental consequences of hypertrophy (e.g., fibrosis and apoptosis) are indifferent to the pressures of natural selection–as happens with many other maladaptive traits found in ageing and cancer–and for these reasons hypertrophy is a common trait in most mammalian species, despite this trade-off.

## Discussion

The main conclusions/deductions of our analysis are as follows: (i) podocyte hypertrophy is not a biophysically or metabolically efficient condition, but a slowly debilitating process. Moreover, as podocyte progenitors do not substantially and substantively contribute to renal repair/regeneration, we maintain that the main evolutionary adaptation for injured glomeruli is hypertrophy, which straightforwardly antagonises functionality and fitness; (ii) tubular cell hypertrophy is not a permanent solution for AKI either; the lack of evidence supporting a supposition that these cells undergo mitochondrial remodelling, which would enable them to optimally perform at a larger size, further weakens the hypothesis that hypertrophy can mediate tissue replacement in reversible tubular injuries. This, in combination with the minor contribution of progenitor cells to tubular recovery (Lazzeri et al., 2018), suggests that the main mechanisms of complete tubular recovery are not related to hypertrophy, but probably dedifferentiation/proliferation and re-differentiation instead. Tubular hypertrophy does indeed exist, but presumably it occurs as a consequence of severe injuries, resulting in a sub-critical number of surviving cells that are not able to regenerate tissue mass through proliferation.

One criticism of our analysis could be the simplicity of the model; cells are conceived of as ideal spheres and we do not take into consideration other parameters, like changes in cell shape and morphology. Nevertheless, the overarching objective was to put the general sense of hypertrophy into a kinetic/energetic perspective for our readers, in a simplified, but conceptually sturdy manner. Other biological elements (like growth factors, receptors, cytokines) are purposely not considered either, as they do not autonomously define the optimal size (Ginzberg et al., 2015). Although further mathematical and biological analyses are needed to definitely explain the role of hypertrophy in glomerular and tubular injuries and repair/regeneration, this new perspective provides a solid theoretical and conceptual platform for explaining the physiological role and evolutionary significance of hypertrophy in kidney injuries.

## Data Availability

The original contributions presented in the study are included in the article/supplementary material, further inquiries can be directed to the corresponding author.
